# Privacy-Preserving Hypothesis Testing for Reduced Cancer Risk on Daily Physical Activity

**DOI:** 10.1007/s10916-018-0930-9

**Published:** 2018-04-04

**Authors:** Hiroaki Kikuchi, Xuping Huang, Shigeta Ikuji, Manami Inoue

**Affiliations:** 1 School of Interdisciplinary Mathematical Sciences, Meiji University, 4-21-1 Nakano, Tokyo, 164-8525 Japan; 2 Strategic Coordination of Research and Intellectual Properties, Meiji University, 4-21-1 Nakano, Tokyo, 164-8525 Japan; 3 ACCESS CO., LTD., 3 Neribei, Kanda, Chiyoda, Tokyo, 101-0022 Japan; 4 Center for Public Health Sciences, National Cancer Center, 5-1-1 Tsukiji, Chuo-ku, Tokyo, 104-0045 Japan

**Keywords:** Privacy, Privacy-preserving data mining, Epidemiology, Hypothesis testing

## Abstract

Privacy preserving data mining for medical information is an important issue to guarantee confidentiality of integrated multiple data sets. In this paper, we propose a secured scheme to estimate related risk of cancers accurately and effectively in a privacy-preserving way. We study models to configure the appropriate set of attributes to reduce risk of identity of an individual from being determined. We examine the proposed privacy preserving protocol for encrypted hypothesis test, using actual cohort data supplied by National Cancer Center.

## Introduction

### Background

Risk factors for cancers have been widely investigated in conventional works. For examples, Cardis et al. [1] at International Agency for Research on Cancer carried out collaborative studies of cancer risks after low doses of ionizing radiation among nearly 600,000 radiation works in the nuclear industry in 15 countries. The result indicates the excess relative risk for cancers other than leukemia was 0.97 per Sv, 95% confidence interval 0.14 to 1.97. They also figure out that excess risk of cancer exists for nuclear workers even at the low doses and dose rate.

However, during these studies of cancers, confidentiality and criticality of privacy information should be considered because of exposure of cancers. For big data mining, integration of multiple data collected via ubiquitous sensors, smart-phones, and portable devices makes epidemiological study more accurate. To achieve accurate data processing as well as privacy preserving, we consider the issues as follows:

**privacy issues of patients**: Given in a confidential dataset, even for medical studies, no cancer patients want to be exposed due to privacy concerns.
**inconsistent identities in multiple datasets**: Proprietary identifiers are used to identify individuals. However, in case of integration of multiple datasets, it is difficult to assume a global identity. In case of integrated datasets with inconsistent identifiers, finding alternatives of identities has been a challenge.

A set of personal attributes, which is used to identify individuals, includes name, address, and telephone numbers, etc. However, models should be studied to configure appropriate set of attributes, especially optimal combination of attributes because of the unavailability of uniqueness of personal attributes.

### Related works

In the conventional works, statistical inference applied to single or multiple datasets has been proposed in many works. Privacy preserving algorithm is required because of the possibility of identifying individual participants by publicly available aggregate statistics, pointed out by Homer et al. [2]. Statistical estimator is studied by Smith [3] and Rakesh et al. [4] and risk-utility has been discussed in Fienberg et al. [5]. Binary hypothesis testing under privacy constraints for large datasets has been studied in Liao et al. [6]. Privacy preserving protocol for radiation data and partitioned data are discussed by Kikuchi, et al. [7] and Vaidya et al. [8].

In order to figure out features of medical data and to clarify correlation between different parameters in a dataset or causal correlation between attributes from different datasets, hypothesis testing supplies an effective statistical inference to determine distribution in a certain dataset, however, in most of conventional works, raw data is used for analysis.

In this work, we propose a new private preserving hypothesis testing protocol, as well as specifying the best combination of significant personal attributions as the quasi-identifier to identify particular user in multiple datasets for statistics inference.

### Our contributions

In this paper, we propose privacy-preserving schemes to estimate the relative risk (RR) of cancers, using the cryptographic protocol *Private Set Intersections (PSI)* to achieve secured epidemiological processing including set intersection for mortality rate, and evaluation of test statistics for hypothesis testing. Confidentiality of data is preserved even after intersection of two subsets.

Our contributions of are listed as follows:

- propose a privacy-preserving protocol for hypothesis testing using a set of personal attributes as quasi-identifiers
- take an experiment of the proposed protocol to estimate relative risk of cancer in terms of quantity daily physical activities

## Privacy-preserving hypothesis testing

### Purpose statement

In this paper, we consider a use case of data analysis toward distributed datasets supplied by different providers.

In case of risk of radiation, suppose party *A* be an agency which maintains lists of workers who are exposed to dose of radiation. This kind of data is available since there are regulations specifying the limit of total annual dose of radiation and employees in nuclear-power stations are supposed to declare the record of dose of radiation in many countries. In Japan, working under more than 50 mSv is prohibited [9, 10]. Party *B* is a hospital for cancers and keeps a dataset of cancer patients.

Both of parties *A* and *B* should keep their datasets *X*_*A*_ and *X*_*B*_ confidential, however, correlation between the risk of cancer and dose of radiation should be contributive and useful for further medical care and research. Thus, a privacy-preserving scheme for confidential computing for distributed dataset is required.

Death rates or *mortality rate* for both datasets are compared to clarify the correlation. The mortality rate is adjusted for different distributions of age groups in both of datasets. Let *X*_*A*,*y*_ be a subset of *X*_*A*_ with increments of 10 years. Then *X*_*A*_ can be partitioned as *X*_*A*_ = *X*_*A*,30_ ∪ *X*_*A*,40_ ∪… ∪ *X*_*A*,80_. The expected numbers of subjects to death can be known as *standardized mortality rate*.

### Relative risk

We examine the risk factors for a disease by dividing participants into two groups with and without exposure in a cohort study. The *relative risk* is defined as the ratio of a diseased member receiving exposure to a diseased member without receiving exposure [12].

Table 1 shows a *contingency table* for a case-control study for cancer as an example, which is a 2 × 2 table of observed frequencies with a sample size *N*. In Table 1, *m*_1_ represents smoking participants and *m*_2_ represents non-smoking participants. *a* and *b* suffer from cancer, while *c* and *d* do not during the investigation. Then the *RR* of smoking is defined as the probability of diseased (cancer) participants in the exposed (smoking) group out of the probability of diseased participants in the unexposed (non-smoking) group as follows.

1 $$\begin{array}{@{}rcl@{}} RR &=& \frac{Pr(\text{cancer}|\text{smoking})} {Pr(\text{cancer}|\text{non-smoking})} \\ &=& \frac{a}{n_{1}}/ \frac{c}{n_{2}} = \frac{a(c+d)}{(a+b)c} \approx \frac{ad}{bc} \end{array} $$

**Table 1:** Contingency table for a case-control study

	Smoking	Non-smoking	Total
Cancer	*a*	*b*	*n*_1_
Noncancer	*c*	*d*	*n*_2_
Total	*m*_1_	*m*_2_	*N*

A RR greater than 1.0 indicates an increased risk of disease in exposed group. Hypothesis test is used to examine the confidence of RR in this paper as follows:

**null hypothesis:** *H*_0_: The proportion of participants who suffer from cancer equals between smoking participants and non-smoking participants;
**alternative hypothesis:** *H*_*A*_: The proportion of smoking participants who suffer from cancer differs from non-smoking participants;

Given the null hypothesis *H*_0_, the expected value *E*_1_ is calculated by multiplying two independent probabilities for each cell of the contingency table that *Pr*(cancer) = *n*_1_/*N* and *Pr*(smoking) = *m*_1_/*N*, as follows:

$$E_{1} = \frac{n_{1}}{N} \frac{m_{1}}{N} N = \frac{n_{1} m_{1}}{N}. $$

We compare the difference between observed frequencies *O*_*i*_ and the expected frequencies *E*_*i*_ in each category of the 2 × 2 cells contingency table using *chi-squared test*. The probability distribution *χ*^2^ is computed as follows:

2 $$\begin{array}{@{}rcl@{}} \chi^{2} &=& \sum\limits_{i = 1}^{2\times 2} \frac{(|O_{i} - E_{i}| -1/2)^{2}}{E_{i}},\\ &=& \frac{ N \left( |ad - bc| \pm N/2 \right)^{2} }{ n_{1} n_{2} m_{1} m_{2}}. \end{array} $$

Where, *χ*^2^ is approximated by a *chi-squared distribution* with (2 − 1)(2 − 1) degrees of freedom. Given a chi-squared distribution with one degree of freedom, the outcome of ${\chi _{1}^{2}} = 3.84$ cuts off the upper 5% of the tail of the distribution.

Alternatively, if employ *χ* is with a normal distribution *N*(0,1), we can test whether

3 $$ \chi = \frac{ \sqrt{N-1} \{ (ad - bc) \pm N/2 \} }{ \sqrt{n_{1} n_{2} m_{1} m_{2}} },  $$

is less than *Z*(0.05/2) = 1.960 with 95% confidence.

### Private set intersection

Private set intersection (PSI) is a cryptographic protocol which allows multiple parties to compute the intersection of their private sets without revealing anything about their sets. A number of protocols have been proposed so far after Freedman, Nissim and Pinkas proposed the first PSI protocol using polynomial expression of sets in [17]. Abadi et. al showed the delegated PSI protocol on outsourced private datasets, which assumes the use in cloud data store in [18]. Among then, the following three works can be used to estimate the size of intersection |*X* ∩ *Y* |, which corresponds to the population of smoker (*X*) suffering from cancer (*Y* ).

#### FNP04 (oblivious polynomial evaluation) [17]

The scheme presented in [17] uses oblivious polynomial evaluation in which elements of set are represented as polynomials *f*(*x*) over a finite field. It is a two-party protocol with one party encoding its elements *x*_1_,*x*_2_,… as the roots of the polynomial and the other party evaluating *f*(*y*_1_),*f*(*y*_2_),… in a privacy preserving way. The evaluation of the polynomial turns to be 0 if and only if *x*_*i*_ = *y*_*j*_ ∈ *X* ∩ *Y*. The drawback of the protocol is the computational complexity. The running cost is proportional to the order of polynomial which equals to the number of elements of the set.

#### SSP (Secure Scalar Product) [16]

Scalar product of two vectors is performed securely using an additive homomorphic public-key algorithm. It is a two-party protocol which can be used by many applications as one of primary building block. The many instances of additive homomorphic algorithms include Paillier encryption [14], Lattice-based encryption, and elliptic-curve cryptosystem. The fundamental version allows vectors of arbitrary values. While, the set intersection requires Boolean vector consisting of 1 or 0 value indicating membership of subsets. Hence, it is too expensive (in terms of computational cost) to evaluate the size of set intersection in privacy preserving manner.

#### AES03 (commutative one-way function) [13]

Agrawal, et. al. used a commutative Pohlig-Hellman cipher [15] and secure hash function as building blocks to construct two-party protocol to compute set intersection. As one of the extension, they also showed the modified protocol that obtains only the size of intersection without seeing the elements of the intersection. The idea of their protocol is that commutative property of two independent encryptions allows to figure out the common element of two subsets. Let *f* and *g* be (symmetric but commutative) encryption privately generated by Alice and Bob, respectively. A common element *x* can be identified by testing that *f*(*g*(*a*)) = *g*(*f*(*a*)) because of the commutative property of encryptions.

We show Algorithm 1 that replaces commutative encryptions *f* and *g* by Pohling-Hellman cipher, defined simply as *f*(*m*) = *H*(*m*)^*u*^ mod *p* and *g*(*m*) = *H*(*m*)^*v*^ mod *p*, respectively. Note that the algorithm is the version of size of intersection only and can be used to determine the elements of intersection by modifying the Step 2 to send the (*H*(*x*)^*u*^)^*v*^ together with the ciphertext *H*(*x*)^*u*^.

Algorithm runs efficiently when the subset is small in the domain of values. Suppose that we have a set of integers ranging from 0 to 100 and subsets of 30 elements for each. In SSP protocol, both parties need iterate public-key encryptions for all possible elements of domain, i.e., 100 times. On the other hand, AES and FNP protocols performs as many as the size of subset, i.e., 30 times. FNP requires polynomial evaluation that runs squared times of the order of polynomial, resulting 30^2^ = 900 computations. Hence, AES has the least computational costs of 30 times and is most appropriate for the application that the intersection is small enough to the domain. 
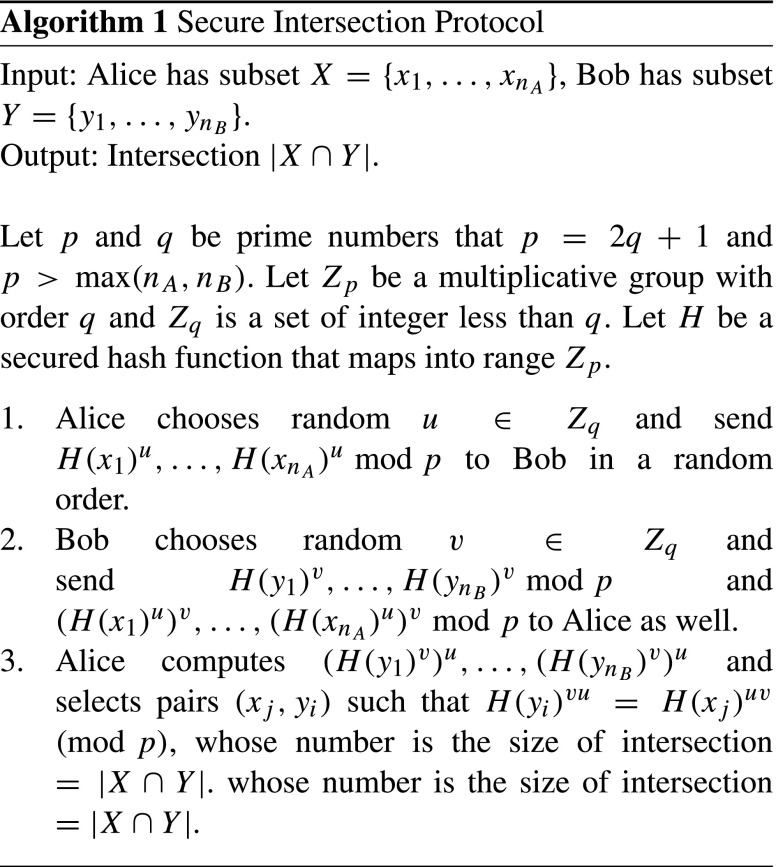


### Hypothesis testing

To model discrete events which occurs infrequently in time series, such as cancer and death, *Poisson distribution* is widely used. Let *X* ($X\in \mathbb {N} \cup \{0\}$) be a random variable that represents the number of occurrences of some events over a given time interval. Let *λ* (*λ* > 0) be a constant that denotes the average number of events in an interval. If the probability that *X* assumes *k* is

4 $$ P(X=k)=\frac{e^{- \lambda} \lambda^{k}}{k!}, $$

then *X* is said to have a Poisson distribution with the rate parameter *λ*.

Suppose we observe *X* = *O* deaths given *E* as the expected number of deaths. We consider a *Standardized Mortality Ratio* (SMR) defined by

5 $$ SMR=\frac{O}{E} = \frac{{\sum}_{j} d_{j}}{\sum q_{j} n_{j}}, $$

where *d*_*j*_ is the observed number of deaths at the *j*-th age interval in the interested condition to be tested, *n*_*j*_ is the general population at *j*-th age interval and *q*_*j*_ is the mean death rate in the *j*-th group. Note that *q*_*j*_ times *n*_*j*_ gives the expected number of deaths at *j*-th age interval.

We wish to determine whether the SMR is close to 1 or not. Namely, if the SMR in workers in nuclear-power station is equal to that of ordinary SMR, the risk of radiation is not significant. Hence, we test null hypothesis

$$H_{0}\;:\; \lambda = E $$

against the alternative hypothesis

$$H_{1}\;:\; \lambda \ne E. $$

If we conduct a one-sided test,

$$\begin{array}{@{}rcl@{}} p &=& P(O|E) + P(O + 1|E) +\ldots, \\ &=& 1-\sum\limits_{j = 0}^{O-1} \frac{E^{j}}{j!} e^{-E} \end{array} $$

gives *p*-value of the test. Employing approximation of Poison distribution when *E* ≥ 5, the test statistic

6 $$ Z=\frac{O-E \pm 0.5}{\sqrt{E}} $$

has an normal distribution *N*(0,1) with mean 0 and the standard deviation 1. Note that 0.5 is the constant. If we conduct a two-sided test, the test statistic satisfying

7 $$ Z=\frac{|O-E|-0.5}{\sqrt{E}}>Z(\alpha / 2) $$

would reject the null hypothesis at the *α* level of significance.

### Privacy search oracle model

Personal attribute may be used to identify individuals. The accuracy of identification depends on type of attributes. For example, attribute sex is 1-bit information to classify the set of individuals into two classes. Birthday has a domain of 365 ways, which is equivalent to log 2(365) = 8.51 bit entropy. Hence, combining sex and birthday could be 1 + 8.51 = 9.51 bit entropy, which could identify 2^9.51^ = 729 individuals in average.

Yasui et. al study some sets of personal attributes in term of entropy in [11]. They proposed Privacy Search Oracle model to quantify the information of attribute based on the statistics of Social Network Services. The population of 120,000,000 individuals is identified uniquely with set of attributes of 27-bit entropy (2^27^ = 134,000,000 > 120,000,000). Table 2 summaries the entropies of some subsets of personal attribute as well as the ambiguity level.

**Table 2:** Entropy of personal attributes

Personal	Entropy [bit]	Ambiguity	Max # of	Description
Attribute			Duplicated IDs	
Name in Chinese char. (Kanji)	27	High	24	Same name can be written in several ways.
Name in Japanese char. (Kana)	N/A	Low	30	Several representations can be unified.
Sex	1	None	61,020	Male or female (1 bit)
Birthday and year	15	None	86	365 days (15 bit)
Mailing address	26	Low	56	Almost unique but several representations in font.
City (ku, machi, mura)	14	High	12,131	Not very unique for historical reason.
Prefecture (states)	6	None	22,336	Unique.
C/A	2	High	N/A	Occasionally specified. not complete attribute.

## Privacy-preserved estimation of reduced cancer risk

### Problem description

Consider Alice is a national cancer center that maintains comprehensive personal attributes for patients of gastric cancer, lung, colon, and so on. In our study, we mainly focus on colon cancer because the risk of colon cancer has been known as significantly correlated to daily physical activity. Alice owns the set of colon cancer as *X*.

Bob is a provider for datasets, indicating interests on personal physical activity. Examples include a sport club that records frequency of exercises for each member, a public health center that periodically investigate personal information of citizens, or a commercial health company that monitors daily physical activity quantities from vital devices. *Metabolic equivalents* (METs) is used to determine quantity daily total physical activity level, based on questionnaires about hours/day in heavy physical works, hours/day in walking, hours/day in sitting, and the days/week in leisure-time sports or exercise [19]. With the METs score, Bob classifies the people into four (*q* = 4) orthogonal classes; Lowest (L), Second (S), Third (T), and Highest (H), specified by four subsets of *U*, *Y*_1_,*Y*_2_,*Y*_3_ and *Y*_4_, respectively.

### Number of people with exact the same names

**Fig. 1:**
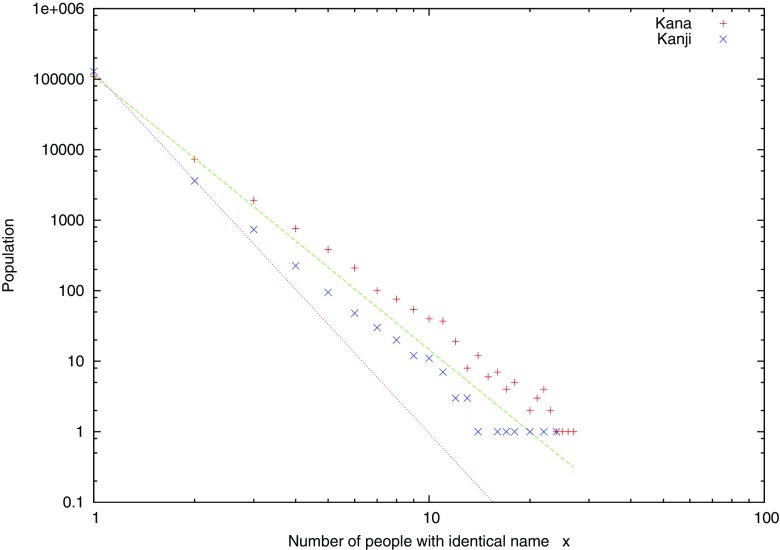
Distribution of Population for numbers of people written with identical name (in Chinese character, labeled as “Kanji”, in Japanese character, labeled as “Kana”)

**Fig. 2:**
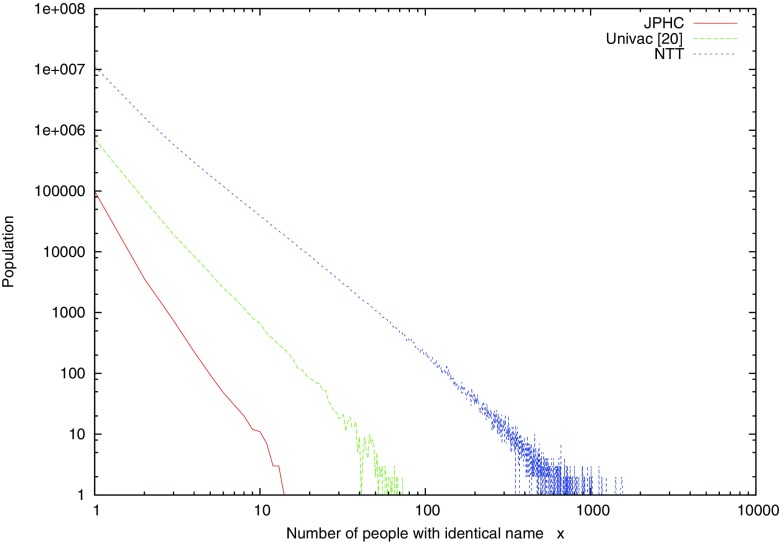
Distributions of population for numbers of people written with identical name in dataset “JPHC”, Univac [20] and NTT

Proprietary identifiers are used to identify individuals for institutes who own datasets. However, in order to join multiple datasets with inconsistent identifiers, alternatives of identifies are necessary. We study a set of significant personal attributes to identify unique individual as a *quasi-identifier*. For example, name attribution is as known as an almost unique attribute, however, there are exceptions that when multiple users have the exact same surname and given name. Figures 1 and 2 shows the population of people in which *x* individuals have the exact same surname and given name in some datasets: JPHC,^1^ Univac [20], and NTT.^2^ Both of vertical and horizontal axis are plotted in log scale. In JPHC, there are about 100 thousand people with unique name (*x* = 1), which becomes about 2 thousand when two people are with the same name (*x* = 2).

Figure 1 shows the number of *x* individuals who have the identical name written in Chinese character (Kanji), and Japanese character (Hiragana).

Based on the observation of the distribution of people with the exact same names, we adapt a mathematical model of *Zipf’s law*, which states that the number of people, *f*(*x*), with the *x*-th order is proportional to 1/*x*.

Accordingly, we have the population of *x* individuals with the same name written in Hiragana as

$$f(x) = \frac{a}{x^{s}} = \frac{110000}{x^{3.87}} $$

where *s* is a constant characterized by the given dataset.

A generalized Zipf’s model allows to estimate the number of people with the same attribute. The total population *D* is given by

8 $$ D = a \sum\limits_{k = 1} \frac{1}{k^{3.87}}. $$

In Eq. 8, we have the constant *a*. For instance, the number of people with not unique name is given from total population in Japan 120 million as *a* = *D*/∑ _*k*= 1_1*k*^3.87^ ) ≃ *D*/1.1 ≃ 109e^6^. Consequently, we estimate that 109 million people have the same name written in Hiragana. Hence, the name in Hiragana is not significant to identify individuals.

### Combination of attributes as a unique identifier

With our Zipf’s model, we quantify the entropy of personal attribute *S*, defined as

$$H(S) = \sum\limits_{k} P(k) \log (P(k))\;\; \text{[bit/symbol]} $$

where *P*(*k*) is a probability of symbol *k*, i.e., value of attribute in *S*. Accordingly, the entropies of name in Kanji and in Hiragana in JPHC dataset of 140,420 records are 14.63 and 13.71 bit/symbols, respectively.

We examine the JPHC dataset with 111,458 records in the same way.^3^ Table 3 shows the number of duplicated (more than two) records for some combinations of personal attributes. For example, a combination of name written in Hiragana and sex is used as the quasi-identifier as option *A*, however, there are 30,180 records which cannot be uniquely identified because more than two records share the exactly the same name and sex. The most common name is shared by 30 individuals.

**Table 3:** Entropies of some combinations of personal attributes

Option	Set of attributes	Entropy	Max #	# of unresolved
		[bit]	duplicated records	records
*A*	Name in Kana, sex	14	30	30,180
*B*	Name in Kana, sex, birthday	30	2	16
*C*	Name in Kana, sex, birthday, state	36	2	12
*D*	Name in Kana, sex, birthday, address	56	0	0
*E*	Name in Kana, birthday, address	55	0	0
*F*	Name in Kana, address	40	2	16
*G*	Sex, birthday, address	42	2	10

According to Table 3, we find options *D* (name, sex, birthday, address) and *E* (name, birthday, address) uniquely identify all individuals in JPHC dataset.

### Proposed scheme

Since identities used by Alice are not consistent with that used by Bob, which considering the combination of multiple datasets, instead of proprietary identities, we use a combination of significant personal attributes, such as names in Hiragana and birthday, as *quasi-identifiers*, which can be computed using secure hash function, e.g., SHA256, as

$$i = \text{Hash}(\text{name } || \text{ birthday } || \text{ address}) $$

where || is a symbol of concatenation. A person who belongs to both datasets *A* and *B* is uniquely identified by the quasi-identifier defined over *U* as the range of secure hash function.

We propose a cryptographic protocol between Alice with *X* and Bob with *Y*_1_,…,*Y*_*q*_ for privacy-preserved relative risk estimation without revealing identities to other parties in Algorithm 2. It uses Algorithm 1 as a sub-protocol.

It aims to compute relative risks in terms of some interested attributes. It is a two-party cryptographic protocol of a party (Alice) with set of (colon cancer) patients and a party (Bob) with questionnaire survey results of patients. Alice and Bob could be the national registry of cancer and local government that conduct user study of citizens. Both parties are not allowed to share the database without consent of all subjects. Instead of sharing, Algorithm 2 allows them to evaluate relative risks of cancer in terms of questionnaire survey without revealing who suffered cancers or the survey results. In our experiment, we are interested in clarifying relative risk of colon cancer in terms of sex and the frequency of daily physical activities. Algorithm 2 also gives the test statistics *χ* to perform hypothesis testing without revealing any personal attribute.

Algorithm 2 runs efficiently even with the big-data studies because the computational cost is not depending on the whole domain size but on the size of interested subsets. 
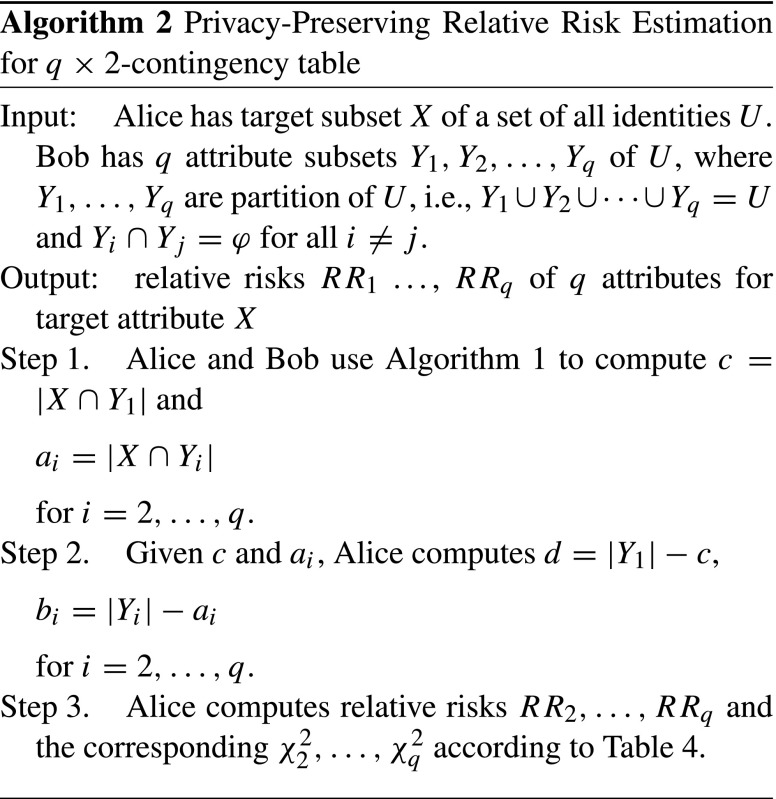

**Table 4:** Contingency table and relative risks with test statistic

	|*X* ∩ *Y*_*p*_|	|*Y*_*p*_ − (*X* ∩ *Y*_*p*_)|	*Y*_*p*_	RR	*N*_*p*_	$ {\chi ^{2}_{p}}$
*Y*_1_	*c*	*d*	*c* + *d*	1.0	–	Reference
*Y*_2_	*a*_2_	*b*_2_	*a*_2_ + *b*_2_	$ \frac {a_{2}}{a_{2}+b_{2}}/\frac {c}{c+d}$	*a*_2_ + *b*_2_ + *c* + *d*	$\frac {N_{2}(a_{2}d-b_{2}c)^{2}}{(a_{2}+b_{2})(c+d)(a_{2}+c)(b_{2}+d)}$
⋮	⋮	⋮	⋮	⋮	⋮	⋮
*Y*_*q*_	*a*_*q*_	*b*_*q*_	*a*_*q*_ + *b*_*q*_	$\frac {a_{q}}{a_{q}+b_{q}}/\frac {c}{c+d}$	*a*_*q*_ + *b*_*q*_ + *c* + *d*	$\frac {N_{q}(a_{q}d-b_{q}c)^{2}}{(a_{q}+b_{q})(c+d)(a_{q}+c)(b_{q}+d)}$

## Experimental evaluation

### Experiment with JPHC dataset

We implemented the proposed protocol and applied it to JPHC Dataset with 99,127 individuals. Table 5 shows the experimental results. For men’s third METs class (*T*), the test statistic is ${\chi ^{2}_{T}} = 6.54$. For chi-square distribution of 1 degree of freedom, since the probability *p* < 0.025 and hence *H*_0_ is rejected. Therefore, daily physical activities in *T*, and *H* for men’s reduces the relative risk of cancer with significant level of confidence.

**Table 5:** Relative risk of colon cancer according to daily total physical activity level

	*X*	|*Y*_*i*_ − (*X* ∩ *Y*_*i*_)|	|*Y*_*p*_|	*RR*	$ \chi ^{2}_{(i)}$
Men *n* = 46, 236					
	(178)	(41,108)	(41,286)		
*L*(16,374)	79	13915	13994	1.00	Reference
*S*(9,594)	36	8229	8265	0.77	1.68
*T*(9,085)	25	7865	7890	0.56	6.54
*H*(11,184)	32	9830	9862	0.57	7.20
Women *n* = 52, 891					
	(130)	(46,330)	(46,460)		
*L*(17,404)	40	14347	14387	1.00	Reference
*S*(13,795)	32	11703	11735	0.98	0.01
*T*(11,865)	32	10283	10315	1.12	0.21
*H*(9,827)	19	8473	8492	0.80	0.61

However, according to the experimental result, test statistics for women are not significant. The possible reason why the METs scores for women are not significant can be assumed that the distribution of ages was skewed, or the other exposure factors such as smoking habit effects.

**Fig. 3:**
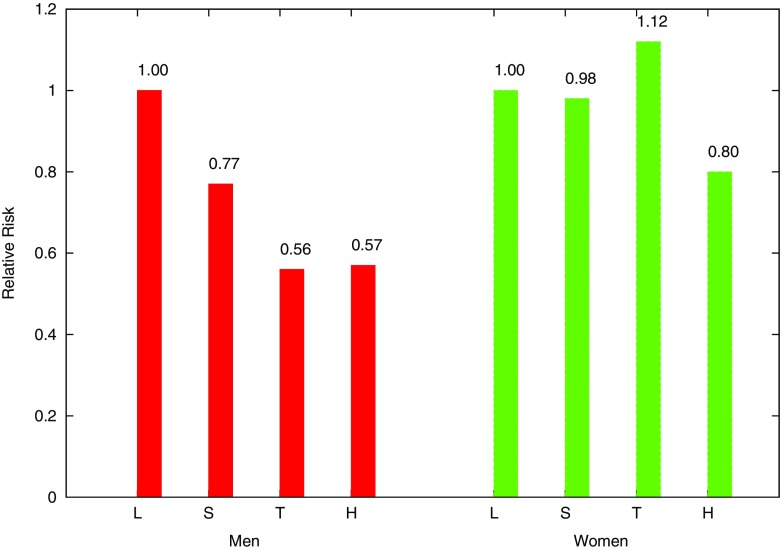
Relative risk of colon cancer for METs estimated in the proposed algorithm

**Fig. 4:**
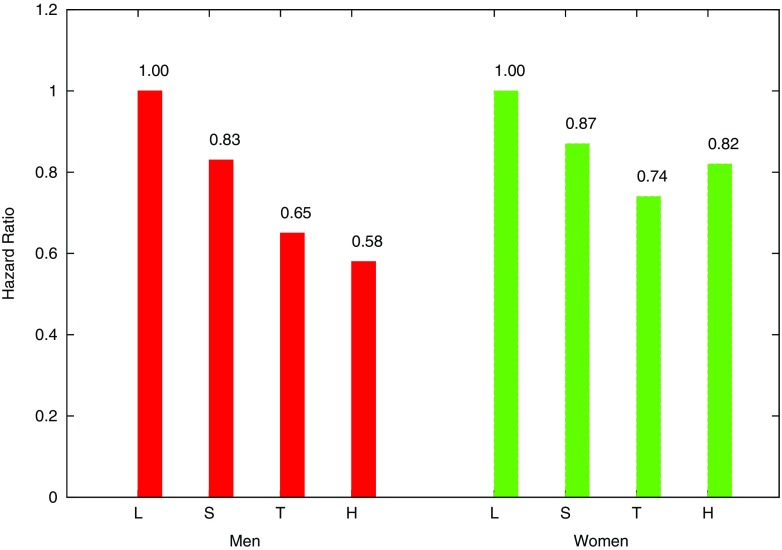
Relative risk of colon cancer for METs estimated in [19]

We compare our result in Fig. 3 to the existing results in [19] in Fig. 4. According to the results, our proposed privacy-preserving protocol achieved a similar behavior correlation between cancer risk and daily physical activities to the conventional work [19] using raw data. Furthermore, the conventional work evaluated relative risk with hazard ratio or odds ratio, while our results are approached more easily in a privacy-preserving way since when dealing with a rare disease, the relative risk can be approximated by the odds ratio.

### Evaluation on performance

**Fig. 5:**
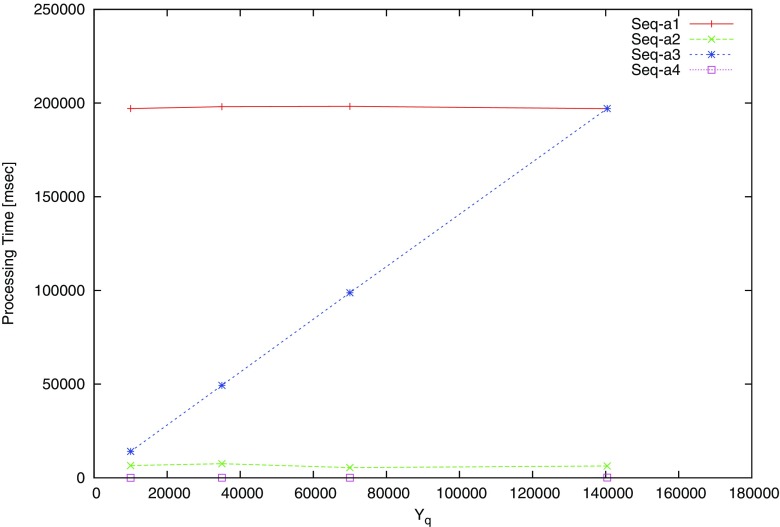
Processing time for dataset size in Alice

**Fig. 6:**
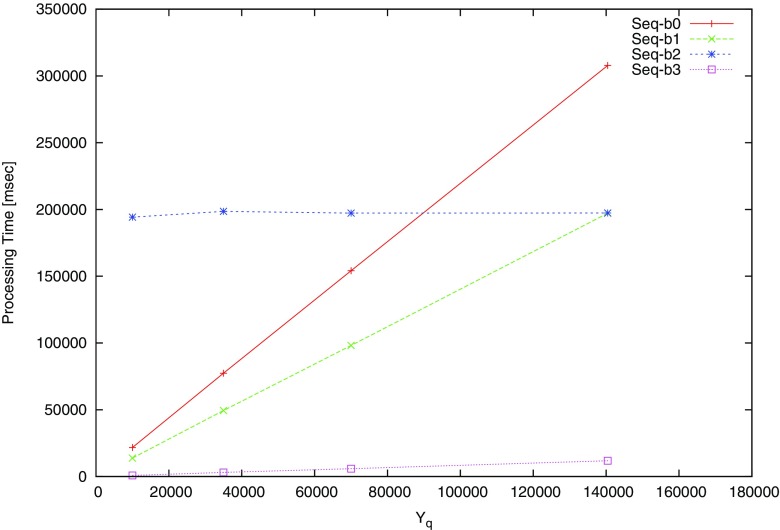
Processing time for dataset size in Bob

We implement and evaluate the performance of the proposed algorithm with JPHC dataset consisting of 140,000 individuals. Table 6 shows the experimental environment to evaluate the performance. Figures 5 and 6 show processing time according to different size of datasets with 10k, 35k, 70k, 140k records. We iterate experiments each for 10 times and record the average processing time.

**Table 6:** Experimental environments

Modulus size |*p*|	2048 bit
Order of *G*	160 bit
Domain of *u*, *v*	160 bit
Application impl.	Scala
SHA-1	Java sphlib
Modulo	Java Big integer
Data Structure	Java HashSet Collection
OS	Ubuntu 12.10 amd64
CPU	Intel Celeron Processor G1610
Memory	4 GB (DDR3 SDRM PC3-10600)
Network speed	46 Mbps (measured values average)

Performance requires a dominant resource for calculation when the modular exponentiation is over than 140k individuals. However, this problem can be solved and the performance can be improved by a distributed computing with multiple machines or parallel computation.

### Evaluation on security

In [13], assuming the random oracle model has no hash collisions, and in semi-honest model, there is no polynomial-time algorithm that can distinguish a random value from *H*(*x*)^*u*^ given *x*.

Summarily, in our use case, data providers are assumed to be *honest-but-curious*, which is known as the *semi-honest* model, that providers own private datasets following protocols properly but trying to learn additional information about the datasets from received messages. It is rational to assume honest-but-curious model because either party may be interested in learning the personal data so that personal data such as name with disease could be dealt in underground market. Malicious model is too strong to assume in two-party case where the malicious party should be excluded easily.

We make the following remarks about the security of the proposed scheme.

#### *Remark 1*

Assuming Decisional Diffie-Hellman hypothesis (DDH), no party learns any element that does not belong to the intersection from the output of Algorithm 1.

#### *Proof*

DDH claims for any element *ginZ*_*q*_, the distribution of 〈*g*^*a*^,*g*^*b*^,*g*^*ab*^〉 is indistinguishable from the distribution of *langleg*^*a*^,*g*^*b*^,*g*^*c*^〉, where *a*,*b*,*c* ∈ *Z*_*q*− 1_. Without loss of generality, assume Alice determines *H*(*y*)^*v*^ for any *y*∉*Y* −−*X* when she has *H*(*x*)^*u*^ and *H*(*x*)^*uv*^ for some known x and u. By replacing *g*^*a*^ = *H*(*x*)^*u*^ and *g*^*ab*^ = *H*(*x*)^*uv*^, she can distinguish *g*^*ab*^ with *g*^*c*^ because she distinguishes *H*(*x*)^*uv*^ with *H*(*y*)^*v*^ from the above assumption. This contradicts to the DDH. Therefore, we have the proof. □

#### *Remark 2*

No party learns any element that does not belong to any intersections for q subsets.

#### *Proof*

It is straightforward from the construction of Algorithm 2. Given *c* = |*X* ∩ *Y*_1_|, there are |*Y*_1_|− *c* possible elements in *Y*_1_, which are impossible to guess with trivial probability 1/(|*Y*_1_|− *c*). Similarly, no information can be learned from *a*_*i*_ = |*X* ∩ *Y*_*i*_|, *b*_*i*_ = |*Y*_*i*_|− *a*_*i*_ for *i* = 2,…,*q*. □

The threat is the risk that malicious parties may figure out the particular individual depending on the chance to identify random numbers in the algorithm. The probability to pick the correct random number is

$$S = \frac{1}{|u|} = 2^{-160}, $$

which is almost infeasible. Thus, the proposed scheme is secure against the malicious party to guess the individual.

### Comparison to conventional works

This paper proposed a privacy-preserving hypothesis testing, which is the novelty of this paper and not achieved by conventional works. Thus, direct comparison is difficult. Instead, we show the relationship between possible building blocks and the proposed two schemes in Table 7. The proposed scheme performs about 360 element per second that is estimated based on the trial implementation. Our scheme performs better than any other protocol based on SSR and FNP because the computational complexity is proportional to the number of element in the subsets.

**Table 7:** Comparison of building blocks

	AES03 [13]	SSR [8]	FNP04 [17]	Proposed
Intersection	Available	No (size only)	Available	Available
Input form	Set	Vector	Set	Set
Complexity	*O*(*n*)	*O*(*N*)	*O*(*n*^2^)	*O*(*n*)
Performance	–	10 dim/s	–	360 elements/s
Relative risk	N/A	N/A	N/A	Yes
Hypothesis test	N/A	N/A	N/A	Yes

### Other applications

PSI protocol is applicable to broad fields not only in cancer risk evaluation but also in enterprise and government. We give some potential applications.

#### Intellectual property and patents

Enterprise having confidential technologies are interested to seek their competitor’s patents. However, it is not clear if their competitor owns unpublished intellectual property that conflict with its private technology. If they share the common technologies, they would like to collaborate or to license each other. If their confidential technologies are disjoint, they want to keep that secret. This can be solved by applying PSI protocol with the set of technical terms of documents. They could make sure whether their unpublished intellectual properties are common or disjoint without revealing confidential document.

#### Epidemiological studies

Epidemiology is study to clarify the outcome of a dose whose effect is not known well. *Dose-Response test* aims to clarify the positive (negative) correlation between an amount of dose and the outcome in a clinical laboratory test. Divided into several groups with same condition, the set of subjects are given a specified amount of doses for each group and observed the responses for the dose. The proposed protocol can be applied to privacy-preserving dose-response test in two parties.

#### Big data study

Link the database of individual tax payments from Tax and Customs office and educational records to reveal the correlations between working in university and their yearly income.

## Conclusions

We have proposed a privacy-preserving hypothesis testing for epidemic studies for calculating relative risk of cancer from distributed providers. The proposed schemes allow independent provides to have confidential datasets to perform computing correlation between any interested attributes. Our experiment shows a close relative risk to conventional work with raw data, which indicating that the daily physical activities reduce a risk of cancer for some experiments in a significant level of confidence.

## Footnotes

1 the Japan Public Health Center-based Prospective Study (JPHC)

2 NTT telephone book 2001

3 It excludes the missing records in some attribute.
